# Recurrent patellar dislocation: treatments and challenges

**DOI:** 10.3389/fsurg.2025.1507362

**Published:** 2025-02-05

**Authors:** Fei Yang, Changshun Chen, Rongjin Chen, Chenhui Yang, Hefang Xiao, Zhiwei Feng, Bin Geng, Yayi Xia

**Affiliations:** ^1^ Department of Orthopedics of Lanzhou University Second Hospital & Orthopedic Clinical Medical Research Center and Intelligent Orthopedic Industry Technology Center of Gansu Province, The Second School of Clinical Medical, Lanzhou University, Lanzhou, China; ^2^ Department of Orthopedics, Nanchong Central Hospital, The Second Clinical Institute of North Sichuan Medical College, Nanchong, China; ^3^ Department of Orthopedics and Trauma Surgery, Affiliated Hospital of Yunnan University, Kunming, China

**Keywords:** recurrent patellar dislocation, surgical strategies, individualized treatment strategies, clinical decision-making, challenges

## Abstract

Recurrent Patellar Dislocation (RPD) is a common knee sports injury, mainly affecting pediatric and adolescent populations, posing a significant challenge in orthopedic clinical practice. Although a variety of treatments have been reported, and many of them have shown good initial results, there is a lack of long-term follow-up results. Each treatment method has its own unique characteristics and limitations, and there is no standardized and unified treatment plan. This article provides a comprehensive review of current treatments for RPD. We believe that regardless of the surgical method used, patellar dislocation should not occur at 0°–90° of postoperative knee flexion and extension, and the range of motion should not be limited. Our ultimate goal is to restore patellar stability and improve lower limb alignment, thereby restoring knee function as much as possible. In addition, future treatment options for RPD are also discussed. In the future, there should be more in-depth research on the risk factors and pathogenesis that lead to recurrent patellar dislocation, as well as more randomized controlled trials focusing on different treatment methods. A comprehensive understanding of these is crucial for implementing preventive measures and developing targeted treatment strategies. The goal of this narrative review is to offer clinicians a deeper understanding of RPD treatment, enhance clinical decision-making skills, and encourage personalized and efficient management of RPD treatment.

## 1 Introduction

Recurrent patellar dislocation (RPD) stands as a significant orthopedic concern, particularly prevalent among the pediatric and adolescent population (1–3). The incidence of RPD in children and adolescents ranges from 23 to 43 per 100,000 persons per year, with the highest prevalence observed in adolescents aged 14–18 years (1, 2). Notably, Gravesen et al. (3) showed that young females aged 10–17 face the highest incidence, underscoring the substantial risk associated with this demographic. Key risk factors contributing to the development of RPD have been identified in recent research. Among these factors, MPFL laxity, skeletal immaturity, trochlear dysplasia, increased patella height, and a history of contralateral patellar dislocation emerge as significant contributors (4, 5). Understanding these risk factors is crucial for both preventive measures and the development of targeted treatment strategies.

Clinically, RPD poses a disabling condition with various associated complications. Articular cartilage injuries, osteochondral fractures, recurrent instability, pain, decreased activity, and the potential development of patellofemoral osteoarthritis are among the notable complications (4, 6, 7). Skeletally immature patients with RPD exhibit high rates of recurrent patellar instability, particularly in cases involving structural abnormalities such as patella alta and trochlear dysplasia (8). Long-term follow-up studies reveal that nearly 10% of these patients experience contralateral dislocation, while 20% develop arthritis within 20 years following the initial dislocation (9). This highlights the importance of early diagnosis and intervention to mitigate long-term complications.

The management of RPD involves a spectrum of treatment modalities, prominently comprising surgical and non-surgical approaches. Surgical intervention, frequently recommended for recurrent dislocations, encompasses procedures such as MPFL reconstruction, trochleoplasty, and lateral release. Notably, the precision of graft placement in MPFL reconstruction emerges as a critical determinant for achieving successful outcomes (10). Conversely, nonoperative treatment stands as the standard approach for initial dislocations, emphasizing rehabilitation and physiotherapy (11, 12). Despite these efforts, recurrence rates remain elevated, underscoring the complexity of the condition. However, the debate between surgical and non-surgical interventions for patellar dislocation continues. While surgical management is linked to a significantly lower risk of subsequent patellar dislocation, it also presents a higher risk of patellofemoral joint osteoarthritis compared to non-surgical management (13). Recent trends in the field highlight a shift towards personalized surgical interventions, tailored to individual patient anatomy and risk factors. This includes a focus on preoperative planning based on specific anatomical abnormalities. Furthermore, the development of predictive models for recurrence risk, incorporating factors such as age, trochlear dysplasia, and skeletal immaturity, plays a crucial role in prognostic counseling and treatment planning (5).

This article systematically reviews the conservative treatment, surgical treatment, and emerging treatment techniques of RPD. It aims to provide insights into the efficacy of surgical and non-surgical interventions, highlight the importance of individualized treatment plans. By synthesizing the latest evidence and trends in the field, the review endeavors to inform clinical decision-making, contributing to the advancement of optimal management strategies for this challenging orthopedic condition.

## 2 Non-surgical treatment of RPD

The non-surgical management of RPD emphasizes the implementation of physical therapy and functional training to address muscle imbalances and enhance patellofemoral joint stability (14). Currently, conservative treatment is considered appropriate for patients experiencing primary acute patellar dislocation, with a achieving favorable functional outcomes. However, a notable percentage of individuals may still encounter recurrent dislocations despite nonoperative interventions (11, 15).

Non-surgical treatment strategies should focus on resolving pain and edema, restoring motion, and strengthening exercises targeting the hip and knee. Rehabilitation protocols include various strategies based on immobilization, weightbearing status, quadriceps exercise type, and alternative therapies. For instance, patients may gradually return to activities with the support of a brace or kinesio tape until preinjury muscle saturation and strength are restored (16). The use of a hinged knee brace or lateral stable brace is also highlighted to enhance knee joint stability, with a specific focus on strengthening the vastus medialis muscle (17). While patient-reported outcomes consistently show improvement following non-surgical treatment, they may not always return to pre-injury levels. A retrospective study by Atkin et al. (18) reported limited physical activity in 58% of patients and found that 55% had not returned to sport after a 6-month follow-up. The recurrence rates of patellar dislocation treated nonoperatively were high and close to the redislocation rates reported in natural history studies (19). Despite the efficacy of non-surgical management, recurrent instability remains a concern, with rates reaching up to 60% in some cases (13), the high rate of recurrent instability highlights the need for improved muscle strengthen strategies and individualized therapy approaches (20). Factors such as osteochondral lesions and recurrent instability may contraindicate nonoperative treatment (21).

In conclusion, while non-surgical treatment of RPD, particularly through physical therapy and functional training, can lead to improved outcomes, there is a notable risk of recurrence. This necessitates the development of more effective and individualized rehabilitation strategies.

## 3 Surgical treatment of RPD

From a treatment perspective, RPD poses a challenge. Conservative strategies predominantly encompass physiotherapy and targeted muscle strengthening, with the overarching goal of enhancing patellar stability. Surgical options are considered for recurrent cases or when conservative treatments fail (14, 15). Surgical treatment aims to reduce of the patella, reconstruct the ligament, or reshape the joint morphology to improve the stability of the patella in the femoral trochlea (22).

### 3.1 The medial patellofemoral ligament (MPFL) reconstruction

The structure of the patellofemoral joint is complex, and its stability depends on the morphology of the joint as well as static and dynamic stability structures. Any anatomical changes (such as defects in the arrangement of the extensor apparatus, dysplasia of the patellofemoral joint, trauma, etc.) may lead to patellar instability. The MPFL is the primary stabilizer to prevent lateral displacement of the patella, providing 50% to 80% inward binding force against the patella sliding outwards (23, 24). Biomechanical studies suggest that the MPFL is the main structural limiting lateral *p* sliding of the patella atellar dislocation at 0° to 30° of knee flexion (25). Xu et al. (24) study showed that MPFL tears occur in up to 90% of patients after a primary patellar dislocation. Notably, 15%–44% of patients with primary dislocation will redislocation after conservative treatment (26). The MPFL reconstruction has good preventive effects, so has been more and more widely used in recent years (27). It is now accepted that the indication for MPFL reconstruction is in patients with recurrent dislocation and no patellofemoral joint deformity or trochlear dysplasia (28). Nelitz et al. (29) showed that anatomic reconstruction of the MPFL that protects the distal femoral physis in skeletally immature patients is a safe and effective technique for the treatment of patellofemoral instability and allows patients to return to sports without redislocation of the patella. In a French multicenter retrospective study, Bremond et al. (30) treated 54 patients with RPD with skeletal immaturity with simple MPFL reconstruction and soft tissue femoral fixation technique, with significant improvements in Kujala, LFPI, Tegner, and NRS scores at a mean follow-up of 2 years. Meynard et al. (31) retrospectively analyzed 113 cases of RPD treated with MPFL reconstruction alone, and the results showed that 91% of the patients recovered their mobility level after surgery, and 67% of the patients returned to the same or higher exercise level as before the injury.

**Figure 1:**
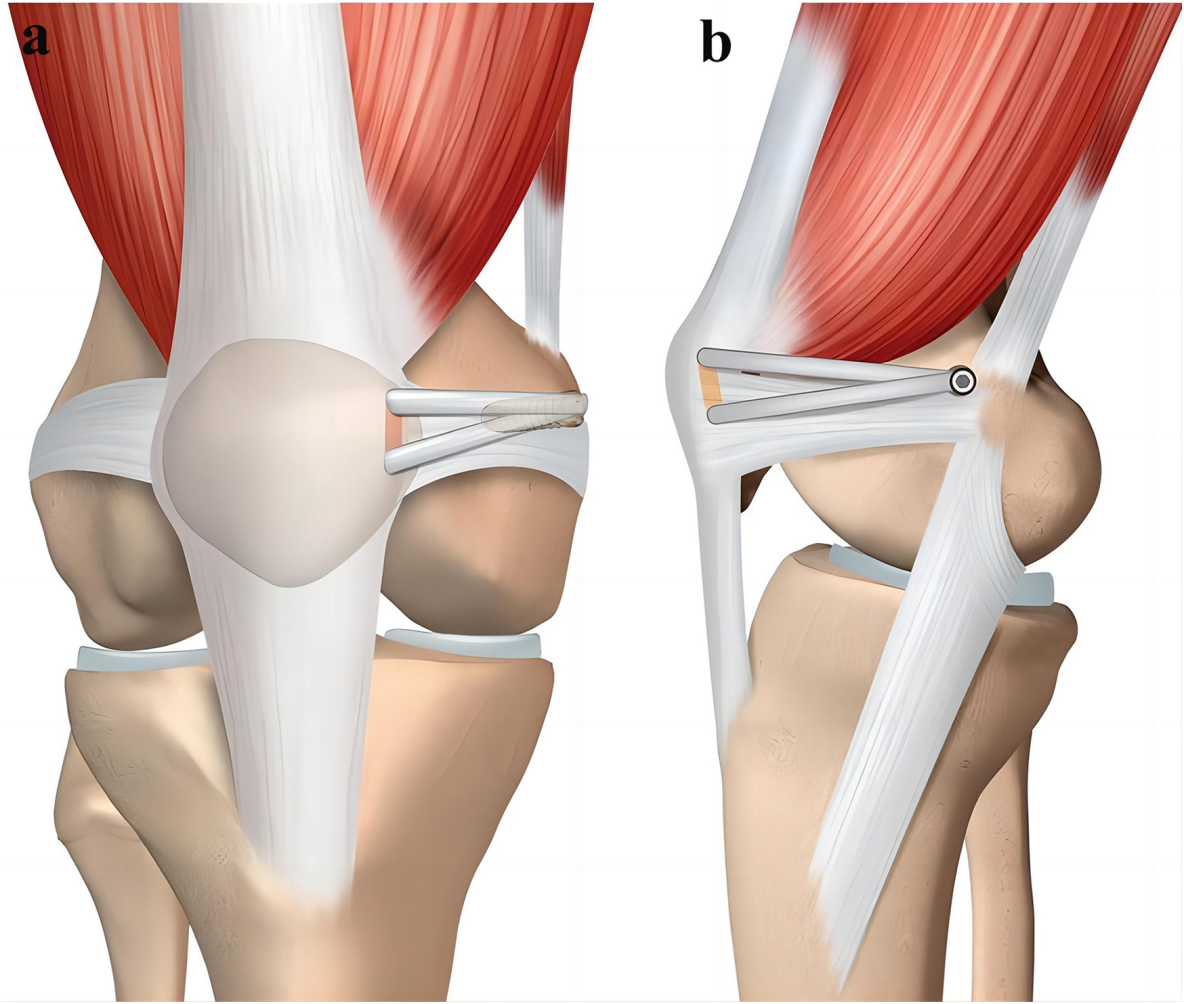
Schematic presentation of the applied MPFL reconstruction. **(a)** Anteroposterior view of the two tunnels in the superior-medial border of the patella. **(b)** Lateral view of the graft secured in the femur [with permission-Ntagiopoulos et al. (33)].

With the deepening of research on the treatment of RPD, the reconstruction techniques of MPFL are also constantly being innovative. Although Rachel et al. (32) suggested that the location of attachment point, type of graft and fixation method are different, each reconstruction technique can better restore patellar stability and knee joint function (Figure 1). However, in terms of MPFL reconstruction, the choice of graft, the selection and fixation of anchor point, the Angle of knee flexion during graft fixation, and the tension of graft fixation are the key factors that determine the success or failure of MPFL reconstruction.

#### 3.1.1 Selection of graft

Graft selection is an important consideration for preoperative MPFL reconstruction, and the ideal graft should have similar biomechanical properties to MPFL (34). At present, it is preferred to select autologous gracilis, semitendinosus or quadriceps tendon, and allografts or synthetic grafts (Table 1).

**Table 1:** Some studies related to MPFL reconstruction with different grafts.

Author	Graft	Results/conclusions	References
Nelitz et al.	Hamstrings	21 patients with immature bone were followed up for an average of 2.8 years, and no re-dislocation occurred	(29)
Schlichte et al.	Hamstrings	A 12-year-old patient was treated with this technique, and no re-dislocation occurred during the follow-up of 2 years	(39)
Ladenhauf et al.	Hamstrings	23 adolescent patients were followed up for an average of 16 months, and no recurrent dislocation was found	(40)
Vavalle et al.	Quadriceps tendons	16 patients were followed up for an average of 38 months, and no recurrent episodes of dislocation or subluxation and no complications occurred	(36)
Fink C et al.	Quadriceps tendons	17 patients were treated with quadriceps tendon graft for MPFL reconstruction. After 12 months of follow-up, the function improved without dislocation recurrence	(41)
Nelitz et al.	Quadriceps tendons	25 adolescent patients were treated. The average follow-up time was 2.6 years, and no re-dislocation occurred	(42)
Vavalle et al.	Quadriceps tendon	16 adolescent patients were treated. The average follow-up period was 38 months, and no recurrent dislocation occurred	(36)
Krishna Kumar et al.	Gracilis	30 patients were treated by MPFL reconstruction with gracilis. All patients were followed up for an average of 25 months with good results and no dislocation recurrence	(43)
Xu et al.	High strength sutures	17 patients were followed up for an average of 12 months, and one patient had recurrence of patellar dislocation and none of the others had serious complications	(24)
Rueth et al.	Allogeneic gracilis tendon	90/101 patients were followed up for an average of 32 months, and the redislocation rate was 0.9% (1/101)	(35)
Husen et al.	Allogeneic tendons	69 patients were followed up for an average of 37.9 ± 12.1 months, and 11 patients experienced a redislocation of the patella, and 1 patient experienced patellar fracture	(37)
Allahabadi et al.	Allogeneic tissues	20 patients (24 knees) were followed up for an average of 5.2 ± 1.7 years, and 3 (12.5%) had recurrent instability, and 1 sustained a patella fracture after a fall	(38)

Rueth et al. (35) considered that anatomic reconstruction of MPFL with gracilis tendon allograft is a safe method for children and adolescents with patellar dislocation with low risk of recurrent instability. Vavalle et al. (36) showed that isolated MPFL reconstruction using an autologous quadriceps tendon, without the need for a bone tunnel, may be a safe, simple, and effective way to treat patellar instability without complications such as patellar fracture, as reported in clinical studies using hamstring grafts. For the same reason, it is also suitable for patients with skeletal immaturity. Olotu et al. (25) suggested that the quadriceps tendon autograft produced improved clinical outcomes with low rates of recurrent postoperative patellar dislocation and the quadriceps tendon remains a suitable alternative for MPFL reconstruction. Husen et al. (37) showed that Bone-preserving anatomic MPFL reconstruction using allogeneic tendons is a safe and effective method for the treatment of patellar instability in patients with skeletal immaturity, independent of patellar height and mantle dysplasia. Be the same, Allahabadi et al. (38) showed that MPFL reconstruction using allogeneic tissue can be performed safely in children and adolescents with good mid-term follow-up results, few complications, and low recurrence instability rate. Xu et al. (24) used a high-strength suture as a graft to reconstruct the MPFL, and the results showed that it could significantly improve the postoperative knee joint stability, with good mid-term clinical efficacy and low incidence of complications.

However, Weinberger et al. (44) showed that autografts may be associated with higher patient-reported outcomes, there is no significant difference in revision rates between autografts and allografts. This indicates that patient factors and allograft processing techniques should be carefully considered when selecting a graft source. In addition, Stephen et al. (45) showed that different graft constructs, such as double-strand gracilis tendon, quadriceps tendon, and tensor fasciae latae allograft, do not significantly differ in their impact on patellofemoral joint kinematics and articular cartilage contact stresses. Another literature shows that the choice of graft does not affect the MPFL reconstruction results, and the surgeon can use either graft at their own discretion (46).

In addition to the selection of grafts from different sources, the choice of single or double bundles for MPFL reconstruction is also the main issue to be considered before surgery. Unfortunately, the choice of single-bundle or double-bundle MPFL reconstruction is still controversial. Wang et al. (47) reported that both single-bundle and double-bundle MPFL reconstruction can better restore patellar stability. However, when the knee flexion was 15°, the force required for 10 mm lateral displacement of the patella in double-bundle reconstruction was higher than that in single-bundle reconstruction. The simultaneous double-bundle reconstruction has an angular synergistic effect to simulate the extensive trajectory of the MPFL in the patella, which allows the patella to have a greater force against the lateral movement of the patella before entering the femoral trochlea at a smaller flexion Angle. However, double-bundle reconstruction also has some disadvantages, such as greater trauma resulting in increased pain and increased risk of patellar fracture.

#### 3.1.2 Fixation of graft

##### 3.1.2.1 Fixation of the patellar end

At present, patella end fixation technology includes transbone tunnel fixation, suture anchor fixation, interfacial screw fixation, etc. (48). The most common fixation method is bone tunnel fixation, and different fixation methods have their own advantages (49).

Transcosseous tunnel fixation is a traditional method of fixation on the patella (48), although the fixation effect is better, it will increase the risk of postoperative patella fracture. Suture anchor fixation and interface screw fixation technology are fixed by inserting internal implant, which has the advantage of reducing the risk of patella fracture, but can not achieve the fixation effect of postoperative tendon-bone healing (50). Niu et al. (51) performed MPFL reconstruction in 38 patients (38 knees) with RPD and found that internal fixation was not only had less bone loss but also more controllable during drilling, which could theoretically reduce the risk of postoperative patella fracture. This technique provides more bone-tendon contact area than suture anchor fixation and interface screw fixation.

##### 3.1.2.2 Fixation of the femoral end

A bias in the fixed position of the graft may result in variable graft laxity or medial overload of the patellofemoral joint. Correct positioning is crucial to reconstructing the MPFL, and the positioning of the patella attachment point is relatively small for (28), while the positioning of the femoral attachment point is a top priority. The study showed that the femoral attachment point is located at (52) about 10 mm from the distal end of the adductor nodule, with three positioning methods: Schottle (53), Stephen (54) and adductor nodule. At present, the most popular method is to use the Schottle method, that is, on the lateral x-ray in the cortex behind the femoral shaft, through the vertical line at the inflection point of the posterior femoral condyle, cortical junction point and Blumensaat line, and the intersection point of the three lines is the femoral attachment point. However, some scholars (55) have proposed that in many cases, it is difficult to obtain standard knee x-ray lateral tablet, resulting in a deviation in the determination of the anatomical position of the femur. Therefore, the imaging method is only a reference method for femoral lateral positioning, and the positioning of the adductor nodule and the surrounding clear anatomical structure may be more accurate. Recent studies have shown that misplacement of the femoral tunnel may lead to graft relaxation, early graft failure, or excessive pressure on the patellofemoral joint leading to early arthritis (56). The low position of the femoral tunnel can lead to graft relaxation in the extension position of the knee and graft tightness in the flexion position, which is clinically manifested as anterior knee pain and knee flexion limitation (57). Repeated flexion and extension reduces the tension of the graft, which leads to early failure and RPD. A high position of the femoral tunnel can lead to excessive tension of the extension ligament, resulting in similar clinical results (57).

Femoral end fixation can be divided into two categories: soft tissue fixation and bone fixation. Although the recurrence rate of dislocation by soft tissue fixation is higher than (42) of bone fixation, it is often used in children and adolescents because it can avoid damaging the epiphyseal plate and has become a reliable choice for patients with patent epiphyseal. Schneider et al. (58) reported that by creating a blind bone tunnel at the femoral end, the graft could be fixed with an interface screw after passing through this tunnel, and this technique had good clinical results. Alm et al. (59) will femoris or subteninosus tendon wound around the adductor tendon and make it attached to the patellar surface, thus improved adductor suspension reconstruction, found that the method although neither need intraoperative x-ray fluoroscopy and internal substance, and can better control the medial patellofemoral joint pressure, but its dislocation recurrence rate is higher, fixation effect is far less than bone fixation. Panagopoulos et al. (60) a reported another method, which is to temporarily fix the graft, then gradually adjust the graft tension, minimize the postoperative instability, and without interface screw interference, making the femoral tunnel closer to the epiphyseal plate, which is particularly important for patients with patent epiphysis undergoing MPFL reconstruction, but is limited to preliminary published data and surgical technical reports.

#### 3.1.3 The knee flexion angle at graft fixation

Establishing the knee flexion angle during graft fixation helps to determine the “tightness” of the graft during knee movement. In a cadaveric study, Lorbach et al. (61) found that although the knee flexion angle did not significantly affect the patellofemoral contact pressure, in the knee, the natural state when the graft was fixed in joint flexion at 60° position. Patel et al. (62), found that patients with graft fixation at knee flexion angles ranging from 20° to 90° had good clinical results and had a low recurrence rate of dislocation. In addition, Thaunat et al. (57) proposed the concept of optimal MPFL isometric, that is, the graft should maintain the isometric state during the flexion of the knee joint from 0° to 30°.

#### 3.1.4 Tension of the graft

In addition to the location and method of the femoral end fixation, the selection of the graft, and the fixation method, clinicians should also pay attention to the tension of the graft during MPFL reconstruction. Inappropriate tension may lead to adverse consequences such as pain and joint degeneration. Stephen et al. (45) showed that proper surgical technique, especially correct femoral tunnel positioning and graft tensioning, is more important than the type of graft used in MPFL reconstruction. How to achieve appropriate graft tension during fixation remains debatable. Philippot et al. (63) showed through a cadaveric study that an ideal fixed graft tension of 10 N. Carnesecchi et al. (64) performed graft fixation in 50 patients with MPFL reconstruction with a tension of 10 N, and found that the treatment effect was relatively ideal. However, Zumbansen et al. (65) showed in a recent biomechanical study that 2 N fixed graft tension is sufficient to restore the patellar trajectory and patellofemoral joint contact pressure to the natural state, while greater tension only increases the medial patellofemoral contact pressure.

### 3.2 The lateral patellar retinaculum (LPR) ligamentolysis

LPR ligamentolysis is suitable for patients with patellofemoral hypertension (66). It can not only correct the patellar alignment, adjust the patellar position, reduce the pressure of the lateral patellofemoral joint, but also relieve the tension of the nerve endings in the LPR (67).

Tan et al. (68) found that simple LPR release in the treatment of RPD could achieve good short-and medium-term results. Verdonk et al. (66) believed that simple LPR release could effectively reduce the lateral patellar high pressure. Woods et al. (69) reported 20 patients with RPD treated by distal lateral tendon release under arthroscopy, the average follow-up was 27 months after operation, and there was no recurrence case, and the knee joint function was significantly improved. However, simple LPR release has certain limitations, which cannot solve the anatomical risk factors leading to patellar instability, such as patella high, trochlear dysplasia, distance from tibial tuberosity to the lowest point of femoral trochlear groove and large Q Angle, etc., and may be a treatment method that is gradually abandoned (68, 70). Kamalapathy et al. (70) reported that the surgical selection of adolescents with patellar dislocation in the United States has changed greatly. In 2010, the clinical application rate of LPR release alone was similar to that of MPFL reconstruction alone, while in 2018, the clinical application rate of MPFL reconstruction was three times that of LPR release alone. Bedi et al. (71) analyzed the biomechanics of whether LPR release was combined with MPFL reconstruction in 8 cadaver, and the results showed that LPR release combined with MPFL reconstruction could attenuate the lateral displacement of the patella. At the same time, the application rate of LPR release associated with MPFL reconstruction also decreased. At present, most scholars believe that LPR release should not be used alone to treat patellar dislocation, but it can be used as a supplement to other surgical methods (72). However, some scholars have found that MPFL reconstruction combined with LPR release may increase the risk of lateral patellar instability (72).

### 3.3 The medial patellar retinaculum (MPR) or joint capsule tightening

**Figure 2:**
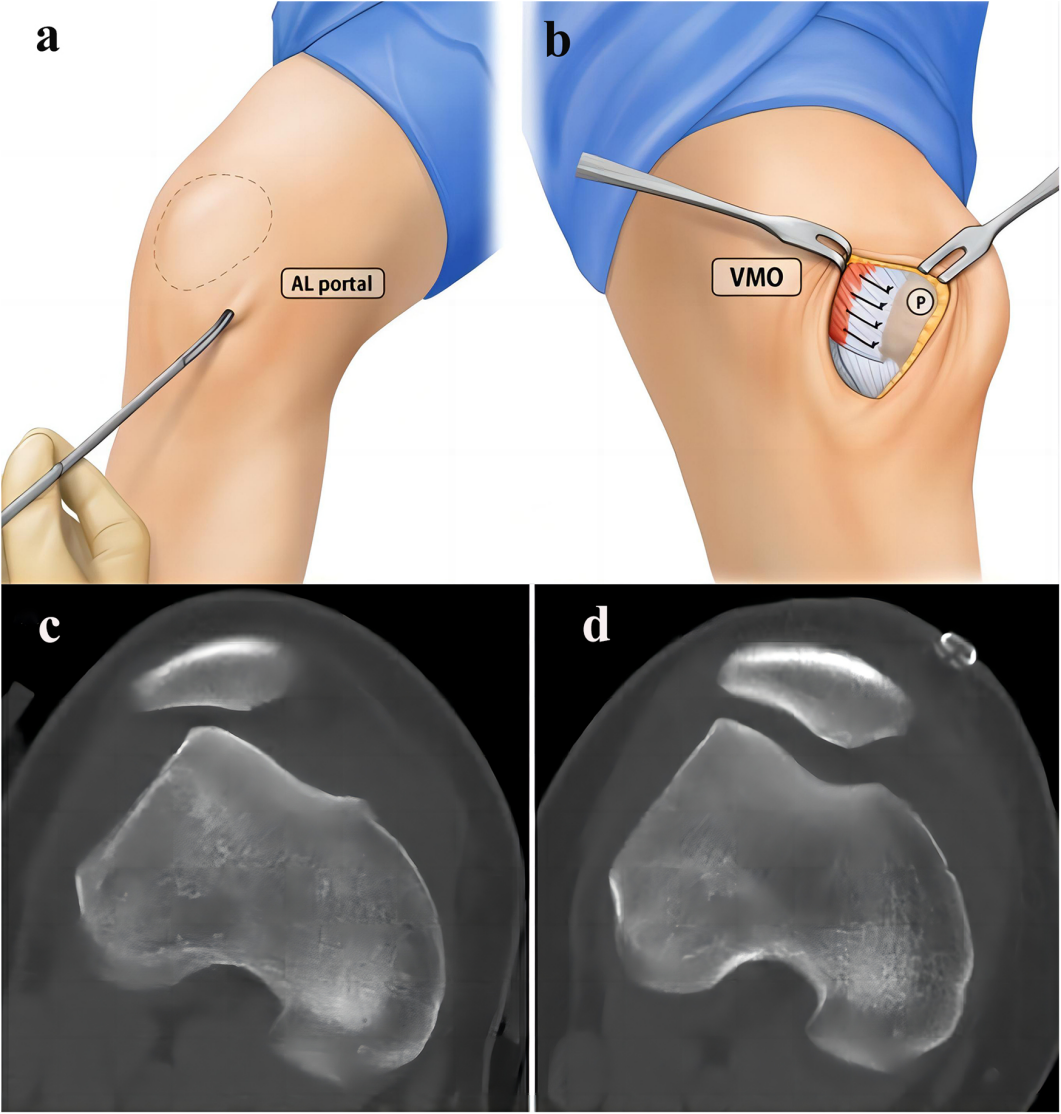
Surgical procedures of the percutaneous LPR release and MPR plication for RPD of the knee. **(a)** The percutaneous LPR release. **(b)** The MPR tightening [with permission-Nha et al. (73)]. A 27-year-old female patient who underwent LPR release and MPR plication. **(c)** Preoperative CT of the knee. **(d)** Postoperative CT showed that congruence angle, patellar tiltangle and lateral patellofemoral angle have been significantly improved [with permission-Wang et al. (78)].

The MPR and joint capsule are also one of the important structures to maintain the stability of the medial patella. MPR tightening or joint capsule tightening is a soft tissue procedure with the advantage that it does not require preparation of patellar or femoral tunnels and has less surgical trauma (73). For patients with RPD and normal bony anatomy, simple medial retinacular tightening surgery under arthroscopy is an effective treatment method (74). However, in general, MPR tightening is often combined with other surgical methods to treat RPD (Figure 2). Schneider et al. (75) reported 18 patients with RPD treated with lateral retinacular release combined with medial capsule tightening, and the Crosby and Insall scoring system had an excellent and good rate of 66. 6% after an average follow-up of 51 months. Coons et al. (76) reported that 53 knees with RPD were treated by monopolar radiofrequency combined with MPR tightening and LPR release under arthroscopy, the average follow-up was 53 months, and the excellent and good rate was 90%, with 5 cases of re-dislocation. However, the better prognosis of MPR tightening combined with LPR release may be related to the fear of re-dislocation after exercise in some patients, and its long-term efficacy may be biased. After long-term follow-up of patients with chronic patellar instability, Schorn et al. (77) found that arthroscopic MPR tightening combined with LPR release was not an appropriate treatment, which may be related to the negative effect of LPR release on both medial and lateral stability.

### 3.4 The MPFL combined with MPTL reconstruction

#### 3.4.1 Anatomic features of MPTL

The medial patellotibial ligament (MPTL) is located in the second layer of the medial patella, the superficial part of the medial collateral ligament and the medial collateral ligament (52) and lateral translation of the patella (79). The MPTL proceeds along the inner interior from its patellar attachment and inserts into the anterior, medial aspect of the proximal tibia, with its tibial attachment point on a newly discovered bony bulge called the medial tibial nodule, on which the MPTL inserts the (34), and formed an angle of about 8.3° with the patellar tendon. On the orthox-ray of the knee, the tibial attachment point of the MPTL is 5. 0 mm distal to the tibial joint line and 5. 6 mm medial to the center of the tibial joint. Furthermore, on the lateral radiographs, the tibial attachment point of the MPTL is 9. 3 mm distal to the tibial oblique line and 16.2 mm ahead of the shaft axis of the tibial bone (34, 79).

#### 3.4.2 Biomechanical effects of MPTL

**Figure 3:**
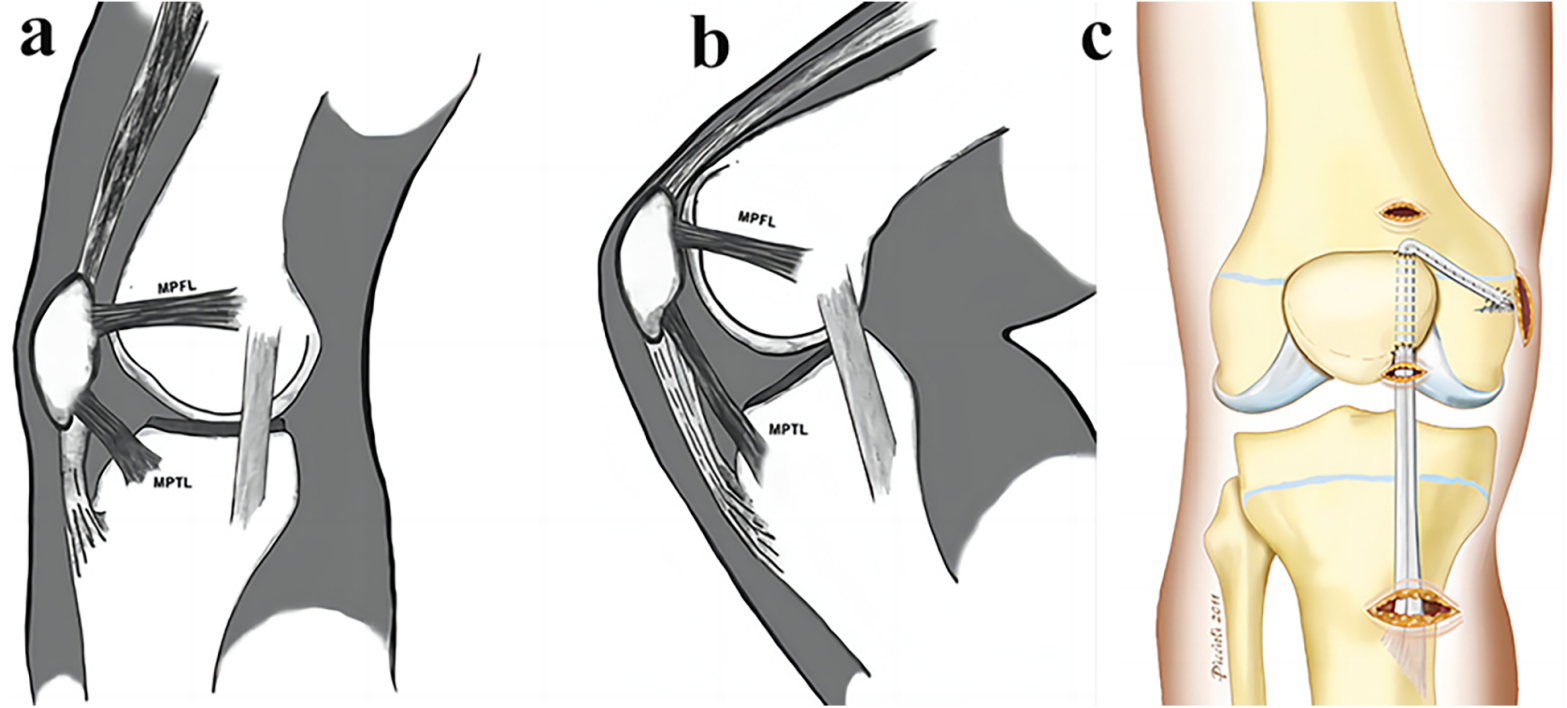
**(a,b)** Anatomical features and biomechanical effects of MPFL and MPTL [with permission-Hinckel et al. (87)]. **(c)** Reconstruction of the MPFL and the MPTL with the hamstrings [with permission-Giordano et al. (86)].

It is precisely because of anatomical structures such as MPTL that it exerts an important biomechanical function. MPFL plays a 23%–80% role in combating the lateral displacement of the patella (34). Thus MPFL binding is only about half of the medial patella binding, and the remaining contribution to medial patella stability comes from MPTL (80). At 0°–30° of knee flexion, the most dominant ligament limiting patellar displacement is MPFL (81). However, when the knee flexion exceeds 45°, and MPTL plays a stabilizing role, The contribution of MPTL to the lateral displacement of the patella can be increased from 26% at knee joint extension to 46% at 90° flexion (63) (Figures 3a,b). Although MPTL is smaller than MPFL in its effect in limiting the horizontal movement of the patella, the absence of MPTL may result in lateral and upmovement of the patella (82).

Therefore, the role of the patella “secondary stabilizer” of the MPTL makes its reconstruction become more important. There are two main mainstream MPTL reconstruction concepts. One is to retain the semitendinosus or thin femoris tibial goose foot stop, lift the other end and fixed at the lower pole of the patella. The other is to separate part of the patellar tendon, where the patella end is motionless and the tibial tuberosity end is fixed to the medial tibia. Both methods simulate the anatomical origin and biomechanical structure of MPTL, with different ways.

#### 3.4.3 MPFL combined with MPTL reconstruction in the treatment of RPD

This technique was first described by Drez et al. (83): First, the semitendinosus and thin ilis tendons are acquired and folded, and their midpoint is partially stitched through a number 2 nonabsorbable woven suture. Then, the median point of the two tendons was sutured to the upper medial side of the patella with a linear bone anchor, and then the end limb of the bicemitoris tendon was sutured to the periosteum at the adductor nodule to achieve MPFL reconstruction. Next, the other end of the bicis tendon was sutured to the tibial periosteum approximately 1.5 cm away from the joint line to achieve MPTL reconstruction, then patella stability was evaluated at full knee extension and flexion for 90°. Brown et al. (84) created such a technique in which a 10 cm incision was made directly in the middle of the patella, and the semitendinosus was found and the tendon stripper was used to release the proximal tendon, while the distal point of goose foot attachment remained intact. A U-shaped tunnel is played in the proximal third of the patella, and one end of the tendon is pulled out through the tunnel to achieve MPTL reconstruction. The knee flexion was performed for 60°, the patella was reduced at the slide to obtain appropriate graft tension, and the tendon penetrated the tunnel end and then sutured to near the medial collateral ligament to complete the MPFL reconstruction. They applied only one tendon and achieved the effect of rebuilding the two ligaments of MPFL and MPTL. The reconstruction technique designed by Ebied et al. (82) was roughly similar to Brown et al, they similarly retained the stop point of hamstring muscle in goose foot, but with a slight difference is that they created a vertical tunnel on the medial side of the patella instead of a U-shaped tunnel. Similarly, the proximal insertion of the gracilis or semitendinosus muscle was released, and then the tendon end was passed through the tunnel and fixed to the femur with the application of intervention screws, thus achieving a ligament reconstruction MPFL and MPTL (Figure 3c). Hinckel et al. (85) used a completely new method to reconstruct the MPFL and MPTL. Through a median knee incision, an 8 mm wide band was separated from the medial part of the quadriceps tendon as a graft, while maintaining its patellar attachment. The other end of the graft was fixed between the medial epiconcondyle and the adductor tubercle to achieve MPFL reconstruction. Then, a 6-mm banded graft was isolated from the medial portion of the patellar tendon, also maintain the attachment of the patella end of the graft and form 20°–25° with the patellar tendon, and then a bone anchor was fixed in the medial tibial MPTL stop to achieve MPTL reconstruction.

Several researchers have tried combined MPFL and MPTL reconstruction and reported encouraging results (82–86). They differed in surgical design, with 5 studies using semitendinosus or gracilis as autografts (82–84, 86). One investigator used another technical alternative, using quadriceps tendon and patellar tendon as grafts, respectively (85). Postoperative follow-up showed that it not only had good results in various functional scores and imaging measurements, but also had a low incidence of re-dislocation and complications.

### 3.5 Femoral trochlea plasty

**Figure 4:**
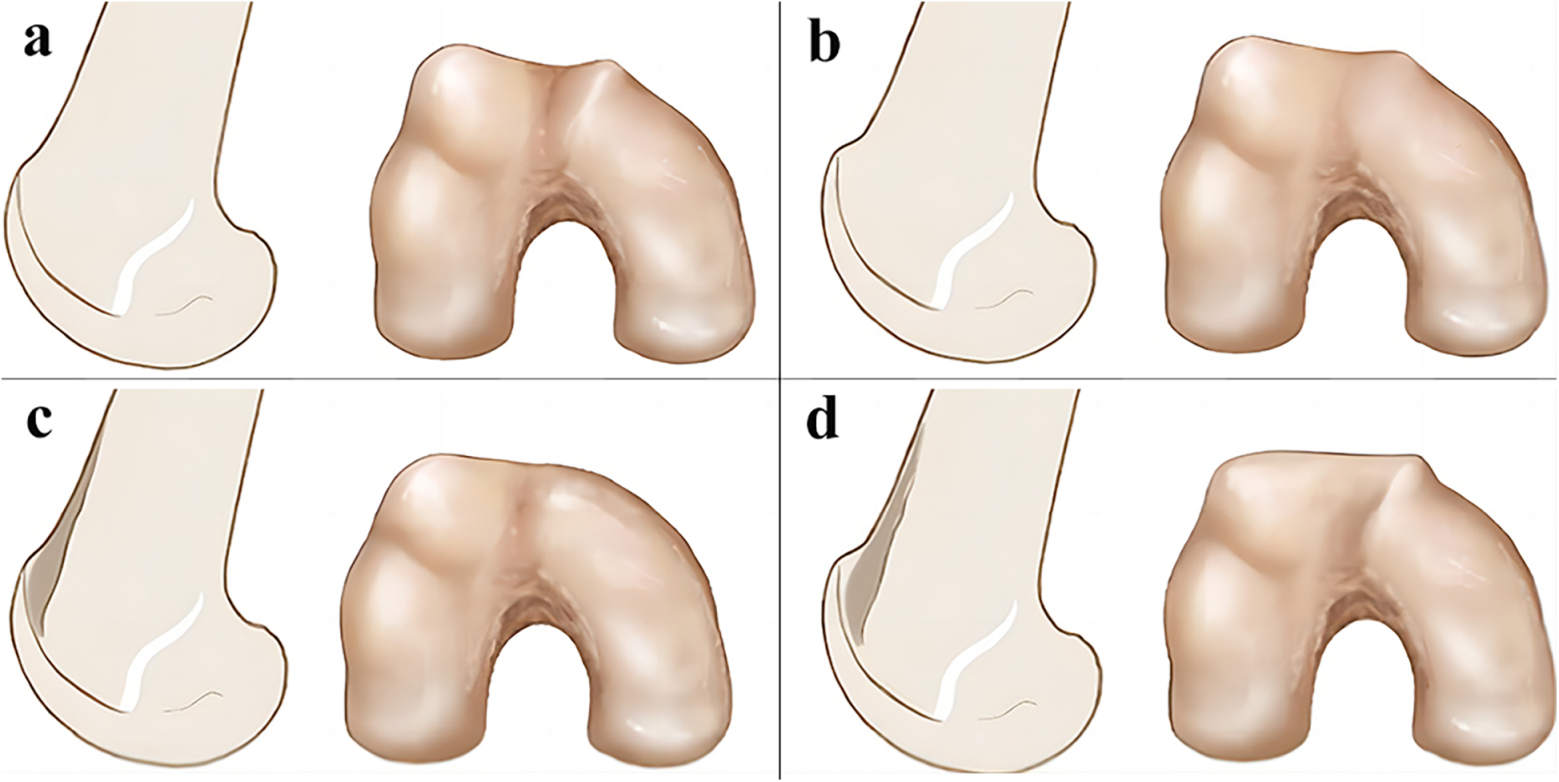
Dejour's classification of trochlear dysplasia in four types: type A with the crossing sign (the groove is flush with the facets), type B with the crossing sign and the supratrochlear spur (“bump”), type C with the crossing sign and the double-contour sign (medial hypoplastic condyle), and type D with all three signs [with permission-Ntagiopoulos et al. (91)].

Femoral trochlear is an important part of patellofemoral movement relationship. RPD is caused by many factors, among which femoral trochlear dysplasia is also one of the important factors, mainly due to the depth and geometric depression of the trochlear groove. Studies have shown that 85% of patients with RPD are accompanied by femoral trochlear dysplasia (88). Dejour et al. (89) proposed that standard anteroposterior and lateral radiographs can be used to classify patients with trochlear dysplasia (Figure 4). At present, scholars believe (90) that the indication of trochlear plasty includes patients with type B, C, and D trochlear dysplasia according to Dejour classification, as well as patients with abnormal patellar tracking caused by trochlear dysplasia. Contraindications include juvenile patients with patent epiphysis and patients with extensive patellofemoral arthritis. Pulley plasty is an intra-articular surgery, which has great trauma to the knee joint and high surgical technical requirements, and is rarely carried out in clinical practice at present. According to the surgical methods, it can be divided into lateral trochlear elevation, trochlear groove deepening, trochlear wedge compression plasty and Bereiter's plasty.

#### 3.5.1 Lateral trochlear elevation

Lateral trochlear elevation was initiated by Albee in 1915, which is the technique of raising the lateral trochlear joint surface to increase its inclination, and restoring its normal anatomical structure through osteotomy and autograft transplantation. In a study conducted by Tigchelaar et al. (92), patients who underwent simple lateral condylar elevation of femur from 1995 to 2002 were followed up for at least 12 years, and the results showed that lateral condylar elevation alone of femur could significantly improve the clinical scores of most patients. However, when performed as a stand-alone procedure, the risk of postoperative patellar redislocation is high, so MPFL reconstruction should be performed at the same time as femoral trocholoplasty. Koeter et al. (93) followed up 19 patients with lateral condylar elevation alone of femur for at least 2 years and found that all patients had significantly reduced pain without recurrence of dislocation. However, the disadvantages of this procedure are that it cannot solve the existing cartilage damage of the patient, nor can it remove the supratrochlear osteophytes, which are common in type B and type D trochlear dysplasia. Moreover, elevating the lateral condyle will increase the pressure in the patellofemoral joint, which may eventually lead to patellofemoral osteoarthritis.

#### 3.5.2 Femoral trochlear groove deepening

**Figure 5:**
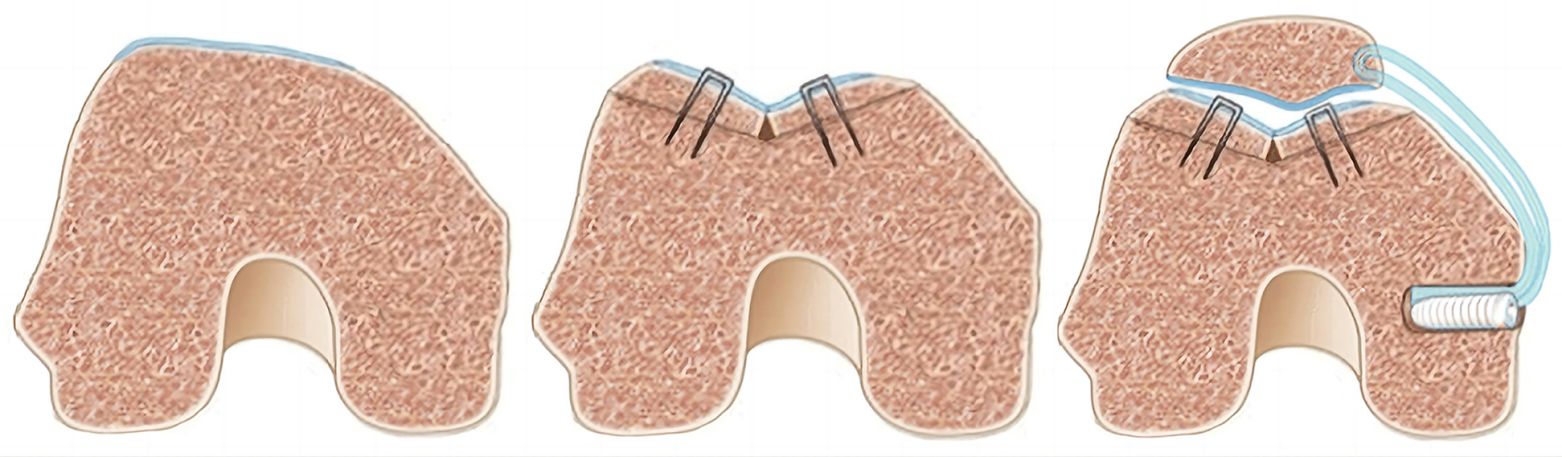
Diagram of sulcus-deepening trochleoplasty (23). (From Maîtrise orthopédique No. 176–2008.).

Masse et al. (94) first proposed the technique of deepening the pulley trench in 1978, and Dejour et al. (95) subsequently improved it (Figure 5). This technique optimizes patellar trajectory by redesigning the trochlear and osteotomy to create a new trochlear groove of normal depth. Davies et al. (96) found that this technique, whether used alone or in combination with other procedures, can improve the knee Kujala score and reduce the recurrence rate of dislocation. Longo et al. (97) compared the effects of various types of femoral trochlea plasty in a retrospective study and found that patients who underwent trochlea groove deepening had the highest postoperative Kujala score. This surgical method can not only remove the osteophytes on the femoral trochlea, but also obtain a more physiological anatomical structure of the femoral trochlea. It is also possible to reduce the TT-TG distance by creating a new femoral trochlear groove, which ultimately saves the patient from additional osteotomy surgery.

#### 3.5.3 Bereiter's trochlea plasty

This technique was proposed by Bereiter et al. in 1994 and is similar to the trochlear groove deepened procedure, with the advantage of avoiding osteotomy through the trochlear articular cartilage. At present, the latest technical concept is to design a new route of the trochlea. After lifting the trochlea cartilage flap, the subchondral bone is repaired with a osteotome, and then the cartilage flap is refixed. Longo et al. (97) in a retrospective study comparing the effects of various types of femoral trochloplasty, found that Bereiter's plasty had the lowest incidence of redislocation and the highest knee range of motion. Hampton et al. (98) followed up 27 patients (31 knees) who underwent Bereiter's trochlear plasty for at least 2 years and found that no patient had recurrent dislocation or needed additional surgery. The advantage of this surgical method is that it can not only remove the osteophytes on the femoral trochlea, but also reshape the femoral trochlea. It is very promising in improving the clinical effect, but it still needs to be confirmed by medium and long-term follow-up (99). At the same time, Thaunat et al. (100) suggest that trochlear plasty should not be performed alone, but should be used in combination with other knee extension device rearrangement. For example, Blond et al. (101) treated 31 cases of RPD (37 knees) with femoral trochlea plasty combined with patellofemoral ligament reconstruction under arthroscopy. During an average follow-up of 12 months, no complications occurred, and 5 cases needed further treatment. Banke et al. (102) used femoral trochlear plasty and MPFL reconstruction to treat 17 cases of RPD (18 knees). After an average follow-up of 2 years, VAS pain scores were significantly reduced, Tegner scores, Kujala scores and IKDC scores were significantly increased, and the surgical results were satisfactory.

#### 3.5.4 Trochlea wedge compression plasty

This technique was first proposed by Goutallier et al. The size and Angle of the wedge to be resected were first determined based on preoperative imaging examination and intraoperative measurements. Then a forward closing wedge osteotomy was performed below the trochlea, and then correction was achieved by gradually applying pressure on the trochlea. The amount of bone removed was just enough to allow the trochlear to be fixed deeper without changing the trochlear groove and finally fixed using cancellous bone screws. Thaunat et al. (100), who performed trochlea wedgeplasty in 19 patients, found that the mean Kujala score, knee injury and osteoarthritis (KOOS) score, and International Knee Scoring Committee (IKDC) score were significantly improved at the last follow-up. Although this technique cannot reconstruct a normal depth of trochlear groove, it can reduce the osteophytes on the trochlear without invading the articular cartilage, which has the potential advantage of reducing the risk of iatrogenic cartilage injury.

### 3.6 Tibial tubercle transfer

The tibial tubercle is the attachment point of the patellar tendon, and its outward displacement affects the patellar tendon force line, thereby changing the “Q Angle” and causing the instability of the patellofemoral joint. Currently, it is believed that the indications for tibial tubercle transfer include Q Angle >20°, TT-TG >20 mm, patella alta (Caton-Deschamps ratio >1.2), patellar instability, patellar instability with inferior and lateral chondromalacia, and patellofemoral joint deformity. However, the epiphysis of the tibial tuberosity was not closed is an absolute contraindication because of the risk of growth arrest and reverse deformity, and this procedure is generally not recommended for patients under 14 years of age (103).

**Figure 6:**
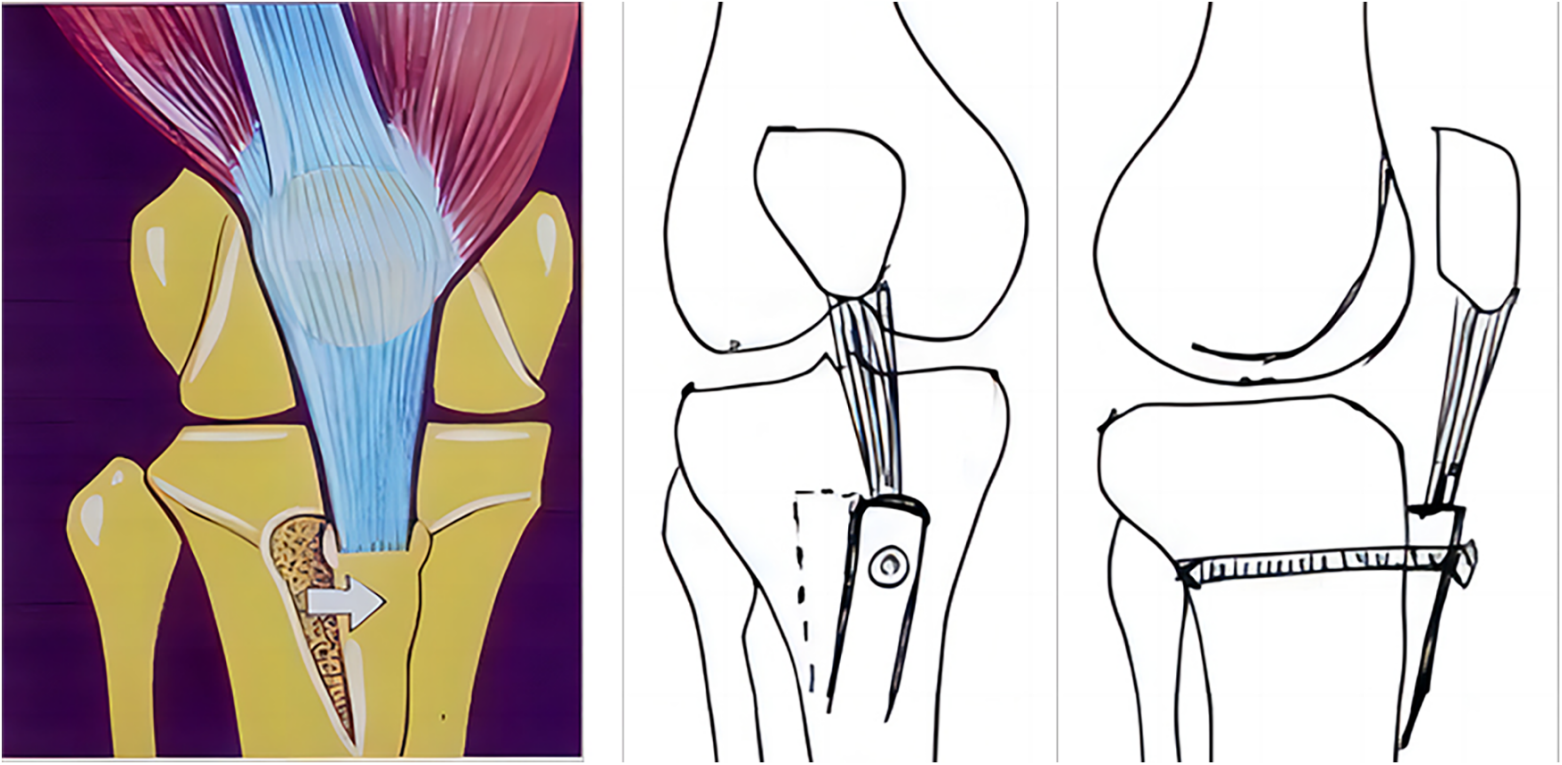
Diagram of tibial tubercle transfer in the treatment of RPD [with permission-Caton et al. (106)].

This technique (Figure 6) was first proposed in 1887 and has since been modified and popularized Based on this, Maquet proposed to elevate the tibial tubercle forward with a bone graft to reduce the patellofemoral joint reaction force, but this process is sometimes complicated by soft tissue injury and wound rupture. Subsequently, Tomatsu et al. (104) proposed the modified Elmslie-Trillat procedure, including medial displacement of tibial tuberosity and lateral retinacular release, omitting the medial joint capsule tightening. Mitani et al. (105) used Elmslie-Trillat osteotomy to treat 31 cases of patellar dislocation of the knee joint, and the knee function score was significantly improved during an average follow-up of 13 years, and it was found that the Kujala score, Q Angle and patellar tilt Angle were improved after operation. This study suggested that this procedure can normalize TT-TG through tibial tubercle displacement, correct the patella alta, reduce its pressure on the patellofemoral joint, and slow down the progression of osteoarthritis. It is a reliable patellofemoral joint surgery.

### 3.7 Osteotomy of the distal femur

The knee valgus caused by the decrease of the mechanical lateral distal femoral angle (mLDFA) and the anterior femoral torsion caused by the increase of the femoral anteversion angle (FAA) can easily lead to the instability of the patellofemoral joint.

The decrease of mLDFA mainly causes patellofemoral joint instability by increasing the “Q Angle”, leading to genu valgum. At present, there are few reports on patellofemoral dislocation caused by genu valgum, and more attention has been paid to knee osteoarthritis caused by genu valgum. The main surgical method is distal femoral osteotomy, including medial wedge closed osteotomy and lateral wedge open osteotomy, both of which can effectively correct genu valgus and achieve good clinical results (107). A large number of studies have shown that both medial wedge closed osteotomy and lateral wedge open osteotomy can achieve good clinical results in the treatment of genu valgus caused by reduced mLDFA (108). Medial wedge closing osteotomy is favored by orthopedic surgeons due to its low risk of nonunion and no need for bone grafting (109). Lateral wedge open osteotomy also has the advantages of easy and controlled osteotomy Angle, safer and simpler lateral surgical approach (110). Meta-analysis of the two surgical methods showed that patients could obtain good knee function after surgery. In the average follow-up of 8.8 years, the survival rate of lateral wedge open osteotomy was 81.5%, and the survival rate of medial wedge closed osteotomy was 90.5% in the average follow-up of 4.5 years (107). In addition, some studies have proposed that the correction of genu valgum by biplanar osteotomy through lateral femoral incision can also obtain satisfactory alignment correction (111). However, most of the current clinical studies are based on distal femoral osteotomy for the treatment of tibial-femoral compartment osteoarthritis caused by valgus knee, and the research on the treatment of patellar dislocation caused by valgus knee is rarely reported, which needs further research.

The FAA of normal people is between 10° and 15°. The excessive torsion of the anterior femur is an important factor leading to the pain of the anterior patellar region, the maltracking of the patella and the dislocation of the patella, and it is easy to be ignored in clinical diagnosis and treatment. Nelitz et al. (112) reported that FAA >25°was a risk factor for patellar instability and recommended surgical correction. Some studies also suggested that FAA ≥30°was a risk factor for the failure of MPFL reconstruction in the treatment of patellar dislocation (113). Surgical correction of the increase of FAA is mainly based on distal femoral derotation osteotomy, the osteotomy plane is perpendicular to the femoral mechanical axis, and the anterior femoral torsion is corrected without changing the lower limb alignment. Studies have shown that distal femoral derotation osteotomy can achieve good clinical results in the treatment of patellar dislocation and prepatellar pain caused by increased FAA (114). Imhoff et al. (115) performed distal femoral derotation osteotomy on 44 knees with an average FAA of 31°, and the FAA was 12° after surgical correction. At the same time, the authors combined the correction of valgus deformity, tibial tuberosity transfer, trochlea plasty and other combined operations. The average follow-up was 44 months, and no patellar redislocation occurred. Zhang et al. (116) compared the patients with de-rotation osteotomy combined with MPFL reconstruction with those with MPFL reconstruction alone, and found that the postoperative MPFL relaxation rate and J sign rate of the osteotomy group were lower than those of the patients with MPFL reconstruction alone.

For patellofemoral joint instability caused by anatomical abnormalities of the distal femur, MPFL reconstruction alone cannot be performed, because the potential lateral force acting on the patella cannot be resolved. In this case, distal femoral osteotomy should be used in combination (117). Zhang et al. (116) compared the difference in the efficacy of MPFL reconstruction combined with and without rotational distal femoral osteotomy, and the results showed that the postoperative Kujala score and Lysholm score of patients with rotational distal femoral osteotomy were significantly higher than those of patients with MPFL reconstruction alone. Jing et al. (118) reported that rotational distal femoral osteotomy combined with MPFL reconstruction is an appropriate method for the treatment of RPD of valgus knee, which can significantly improve the knee joint function and imaging performance in a short period of time.

## 4 Emerging treatment techniques of RPD

The RPD is a common clinical disease, especially in young people. Its treatment and rehabilitation are still difficult problems for surgeons to overcome. Notably, Smith et al. (119) have conducted a comprehensive examination of the comparative effects of surgical and non-surgical interventions for the treatment of the RPD. Their study contributes valuable insights, emphasizing the existing uncertainty surrounding the superiority of surgical approaches over non-surgical methods. This underscores the pressing need for more rigorous research endeavors in this domain to elucidate optimal treatment strategies and enhance clinical outcomes.

### 4.1 Arthroscopic treatment of RPD

With the continuous progress of medical technology, arthroscopic minimally invasive surgery has been developing. The treatment of RPD under arthroscopy is one of the research hotspots in recent years. Using arthroscopic techniques, the surgeon can directly visualize the patella position and perform reduction by minimally invasive surgical means. In addition, the use of arthroscopic techniques allows repair of the soft tissues around the knee joint, including ligament and tendon repair, which helps to enhance the stability of the patella and reduce the risk of re-dislocation. This surgical technique has the advantages of less trauma and faster recovery than traditional open surgery.

Roth et al. (120) used arthroscopic lateral retinacular release to treat 27 cases of RPD (33 knees). After an average follow-up of 4.5 years, Lysholm and Kujala scores were significantly improved, which was conducive to the recovery and improvement of knee joint function. Ali et al. (121) used arthroscopic proximal alignment adjustment to treat 37 cases of RPD (38 knees), after an average follow-up of 51 months, the excellent and good rate reached 78%, and the subjective symptom improvement rate reached 89%, with fast postoperative recovery and high cure rate. Blond et al. (101) treated 37 cases of RPD by femoral trochlear plasty combined with patellofemoral ligament reconstruction under arthroscopy. The average follow-up period was 12 months. There were no complications. Ji et al. (122) introduced a new technique of medial plication using an arthroscopic technique to treat patellar instability in adolescents and used this technique to treat 19 cases of acute patellar dislocation. During a mean follow-up of 3 years, no recurrence of patellar instability was found and the knee range of motion recovered well. Hu et al. (123). introduced an arthroscopic femoral tunnel insertion technique during MPFL reconstruction, which is especially suitable for obese patients and allows minimally invasive tunnel insertion without exposure to x-rays.

In conclusion, advancements in arthroscopic techniques and tailored surgical interventions significantly improve the outcomes for patients with RPD. The choice of technique often depends on individual patient factors, including age, severity of dislocation, and associated injuries.

### 4.2 Biological therapies

Recent research has made significant progress in developing genetic and molecular-based treatment strategies for RPD. It emphasizes genetic and molecular-based therapeutic strategies, the application of stem cells, gene therapy and other methods, to promote the regeneration and repair of cartilage and ligament. For example, Song et al. (124) a case report demonstrated that implantation of human umbilical cord blood-derived mesenchymal stem cells (hUCB-MSCs) in a 15-year-old male with a large patellar cartilage defect due to patellar dislocation led to significant improvement. This included good scores on various scales and MRI evidence of cartilage regeneration 18 months postoperatively, suggesting the potential of hUCB-MSCs in repairing large patellar cartilage defects. Chan et al. (125) a study revealed a genetic component in RPD, showing a balanced translocation of chromosomes 15 and 20 in several family members with recurrent dislocations. This highlights the potential genetic underpinnings of the condition.

### 4.3 Individualized treatment strategies

Liebensteiner et al. (126) a study highlighted the importance of customizing surgical treatment to the patient's specific anatomical pathologies leading to patellar instability. This approach acknowledges the diversity of surgical groups in previous studies and emphasizes individualized treatment strategies. This personalized approach allows for selecting the best surgical option, addressing the specific needs and conditions of each patient.

In addition, with the development of bioinformatics technology, optimizing the treatment plan based on individual differences of bioinformatics and exploring how bioinformatics methods play a role in individualized treatment decisions are also one of the hotspots in the current individualized treatment of RPD For example, Ling et al. (127) development of a multivariable model based on individual risk factors, such as age, history of contralateral patellar dislocation, skeletal immaturity, and lateral patellar tilt, to guide the management of patients with RPD. This model aims to reduce short-term disability and the long-term risk of patellofemoral arthritis from repeated chondral injury. Hing et al. (128) demonstrated the use of computational approaches to compare preoperative patellofemoral joint stability with postoperative stability in various simulated procedures. In summary, bioinformatics and computational methods are contributing significantly to the improved understanding and management of RPD, aiding in the development of predictive models, patient-specific treatment strategies, and providing insight into surgical vs. non-surgical interventions.

## 5 Discussion and outlook

### 5.1 Current therapeutic options and their limitations for RPD

Although there are a variety of treatment measures for RPD at present, each with specific characteristics and limitations. To obtain satisfactory results, it is necessary to conduct detailed clinical evaluation and auxiliary examination of the patient, and select the appropriate treatment according to the patient's anatomical structure and potential pathological conditions.

Acute primary dislocation of the patella is usually managed conservatively, especially in adolescents without obvious anatomical abnormalities or in elderly patients with high surgical risk (129). Some researchers believe that age is one of the key factors affecting the treatment choice of primary patellar dislocation. Chen et al. (130) found that surgical and conservative treatment produced similar clinical outcomes when patients with primary patellar dislocation were ≤20 years of age. Another study has also shown that conservative treatment is the first choice for children and adolescents with a first patellar dislocation in the absence of significant anatomical abnormalities (131). However, a recent study found that early surgical intervention in pediatric patients with a first patellar dislocation is superior to conservative treatment, which is more beneficial to improve clinical symptoms and reduce the rate of re-dislocation (132). The optimal management of a first patellar dislocation remains a subject of debate, especially in juvenile patients (133). Therefore, further high-quality research is needed to provide more definitive guidance.

Surgical intervention is the treatment of choice for recurrent lateral patellar instability. Surgery should be considered for first time lateral patella dislocations with osteochondral fractures or underlying anatomical risk factors (134). Patients with MPFL damage without bony deformity can choose anatomical MPFL reconstruction. Patients with Dejour classification of type B and type D trochlear dysplasia can be treated with trochlear groove deepening or Bereiter's trochlear plasty. Patients with patella alta, TT-TG >20 mm and patellofemoral joint deformity can choose the modified Elmslie-Trillat procedure. It is worth mentioning that despite the high complication rate, MPFL reconstruction is still the most popular method. Some studies (29) have pointed out that although patent epiphysis is a contraindication for many bone surgery, MPFL reconstruction is safe for patients with patent epiphysis. For patients with Dejour type A trochlear dysplasia and TT-TG values exceeding a certain range of normal values, MPFL reconstruction can reduce the risk of dislocation caused by other risk factors to a certain extent. However, for patients with a large degree of MPFL deformity, simple MPFL reconstruction obviously cannot solve all the problems, at this time, combined with femoral trochlear plasty, tibial tuberosity transfer and other bone surgery is necessary. At present, these surgical methods have achieved relatively ideal clinical results, but they are only short—and medium-term results, and lack of long-term follow-up confirmation. Moreover, there are still great controversies about the location of the femoral end of MPFL reconstruction, the indication of trochlea plasty, and the best position of the tibial tuberosity, which need to be further solved.

Interestingly, a recent study showed that in patients experiencing primary or recurrent patellar dislocation, strength deficits in the quadriceps femoris were common in knee extension of the affected limb, regardless of whether they were treated surgically or conservatively. This deficit may persist even with up to three years of follow-up after injury (135). This study further highlighted the important role of quadriceps strength training in the rehabilitation of patients with patellar dislocation. On the one hand, quadriceps strengthening is important to achieve and maintain proper stabilization of the knee joint (136). Smith et al. (137) showed that strengthening of the vastus medialis oblique muscle, an important medial stabilizer of the patellofemoral joint, significantly reduced quadriceps femoris deficit. However, other investigators have argued that quadriceps strengthening should be accompanied by strengthening of the core and hip muscle complexes, as strengthening these muscles is considered important for safe return to sport (138, 139). On the other hand, increased quadriceps strength may protect the patellofemoral joint from cartilage degradation (140). Studies have found that strengthening quadriceps strength in patients with patellar dislocation can reduce the incidence of patellofemoral arthritis (141).

### 5.2 Future directions and challenges of arthroscopic treatment of RPD

Although with the continuous progress of medical technology, more and more surgeons choose to use arthroscopy to treat RPD. Arthroscopy, in comparison to traditional open surgery, presents advantages such as reduced trauma and accelerated recovery. While these advancements are promising, the landscape of arthroscopic treatment for RPD remains intricate, necessitating comprehensive research to address existing challenges and explore future avenues.

First, More studies are needed to verify the long-term efficacy, complications and risk assessment of arthroscopic treatment of RPD. Then, according to the characteristics and clinical manifestations of different patients, future research can explore the individualized arthroscopic treatment plan, including the customization of surgical techniques and postoperative rehabilitation plan, so as to improve the treatment effect and reduce the incidence of complications. In addition, the improvement and innovation of arthroscopic surgical techniques will be the focus of future research. It includes the improvement of surgical instruments, the innovation of surgical techniques, and the exploration of new treatment methods to improve the safety and effectiveness of surgery. More importantly that to study the role and effect of combined arthroscopic procedures, such as lateral ligament release or tibial tuberosity osteotomy. To determine which patients would most benefit from these additional surgical interventions in terms of patellar stability and knee function.

In conclusion, the trajectory of future research in arthroscopic treatment of RPD will emphasize individualized treatment, long-term efficacy evaluation, postoperative rehabilitation, prevention of recurrence, combined arthroscopic surgery, and continual improvement and innovation of surgical techniques. Overcoming challenges related to long-term follow-up, implementing individualized treatment plans, providing guidance in postoperative rehabilitation, and refining surgical techniques are pivotal for the advancement of arthroscopic interventions in orthopedic practice. The collaborative efforts of researchers, clinicians, and technology developers will play a pivotal role in shaping the future landscape of arthroscopic treatment for RPD.

### 5.3 Potential role of bioinformatics approaches in the treatment of RPD

Bioinformatics methods may play an important role in personalized treatment strategies for RPD in the future. Bioinformatics is an interdisciplinary subject that uses mathematics, statistics, and computer science to study biological problems. It can provide important information and guidance for individualized treatment through the analysis of individual genome, proteome, morphological parameters and other biological information data (142, 143).

First, the morphological parameters of femur and patella were extracted from imaging data, and the prediction model of patellar dislocation/instability was constructed using bioinformatics methods, which could provide a basis for specific rehabilitation training programs and individualized treatment (144, 145). Second, through the analysis of genomic data of patients, genetic variants and gene mutations associated with RPD can be found, so as to provide a basis for individualized treatment (146). For example, genetic variants associated with patellar stability can be found to provide guidance for the development of personalized surgical protocols. In addition, through the analysis of patient epigenomic and proteomic data, the epigenetic changes (such as DNA methylation), changes in protein expression level and modification status related to patellar dislocation can be found, thus providing a basis for individualized treatment. For example, proteins or epigenetic changes related to cartilage repair and muscle stability can be identified to guide the development of postoperative rehabilitation programs (147).

In summary, tthe application of bioinformatics methods can realize the formulation of personalized treatment plans for patients with RPD, so as to improve the treatment effect, reduce the incidence of complications, and provide patients with more accurate and effective treatment plans. Therefore, bioinformatics approaches have a potentially important role in the personalization of treatment strategies for RPD in the future.

## 6 Conclusion

At present, all surgical methods have shortcomings, and a standardized and unified treatment plan has not been formed. No matter what kind of operation is used, there should be no patellar dislocation during the flexion and extension of the knee joint from 0° to 90° after operation, and the range of flexion and extension should not be limited. Our ultimate goal is to restore the patellar stability and improve the lower limb alignment, thereby restoring the knee joint function as much as possible. In some cases, a single surgical procedure can solve the dislocation, while in some cases of severe dysplasia, combined surgery should be performed. For patients with severe genu valgum or abnormal femoral rotation, if osteotomy is considered, the osteotomy Angle and rotation Angle should be planned in detail before operation. In the future, more prospective studies should be conducted and more in-depth studies on the pathogenesis of patellar dislocation should be carried out to explore more effective treatment programs, actually solve the clinical problems and relieve the pain of patients.
